# GALNT7 promotes the malignant progression of gastrointestinal stromal tumors by regulating KIT O-GalNAc glycosylation

**DOI:** 10.1093/pcmedi/pbag016

**Published:** 2026-05-27

**Authors:** Jiahao Liu, Sihan Wu, Yunfei Wang, Ge Zhang, Jiehan Li, Zhen Wang, Yuhan Yin, Hao Liu, Sanfei Peng, Yang Fu

**Affiliations:** Department of Gastrointestinal Surgery, the First Affiliated Hospital of Zhengzhou University, Zhengzhou 450052, China; Department of Gastrointestinal Surgery, the First Affiliated Hospital of Zhengzhou University, Zhengzhou 450052, China; Department of Gastrointestinal Surgery, the First Affiliated Hospital of Zhengzhou University, Zhengzhou 450052, China; Department of Gastrointestinal Surgery, the First Affiliated Hospital of Zhengzhou University, Zhengzhou 450052, China; Department of Gastrointestinal Surgery, the First Affiliated Hospital of Zhengzhou University, Zhengzhou 450052, China; Department of Gastrointestinal Surgery, the First Affiliated Hospital of Zhengzhou University, Zhengzhou 450052, China; Department of Gastrointestinal Surgery, the First Affiliated Hospital of Zhengzhou University, Zhengzhou 450052, China; Department of Gastrointestinal Surgery, the First Affiliated Hospital of Zhengzhou University, Zhengzhou 450052, China; Department of Gastrointestinal Surgery, the First Affiliated Hospital of Zhengzhou University, Zhengzhou 450052, China; Department of Gastrointestinal Surgery, the First Affiliated Hospital of Zhengzhou University, Zhengzhou 450052, China

**Keywords:** gastrointestinal stromal tumor, O-GalNAc glycosylation, GALNT7, KIT, malignant progression

## Abstract

**Background:**

Gastrointestinal stromal tumor (GIST) is the most common mesenchymal tumor of the gastrointestinal tract and is mainly driven by activating *KIT* or *PDGFRA* mutations. Although tyrosine kinase inhibitors (TKIs) improve outcomes, primary and acquired resistance remain major challenges, especially in high-risk and wild-type GIST. Protein O-linked N-acetylgalactosamine (O-GalNAc) glycosylation regulates protein stability and signaling, but its role in GIST remains unclear.

**Methods:**

Bulk RNA-seq, proteomic, and single-cell RNA-seq data were integrated to identify O-glycosylation-related programs and key glycosyltransferases in GIST. Functional assays in GIST-T1 and GIST-882 cells, together with xenograft models, were performed to assess the effects of GalNAc-transferase 7 (GALNT7). GALNT7–KIT interaction, KIT O-GalNAcylation, and protein stability were examined by co-immunoprecipitation, VVA lectin blotting, confocal microscopy, and cycloheximide chase assays. Benzyl-α-GalNAc was evaluated as an O-glycosylation-targeting strategy *in vitro* and *in vivo*.

**Results:**

O-glycosylation signatures were enriched in high-risk GIST and correlated with pathological risk. High O-glycosylation scores co-segregated with elevated copy-number variation in a fibroblast-like malignant cell population. *GALNT7* was identified as a hub gene, upregulated in GIST, and associated with poor progression-free survival. GALNT7 promoted GIST cell growth, migration, and xenograft formation. Mechanistically, GALNT7 interacted with KIT, catalyzed its Tn-antigen O-GalNAcylation, increased KIT protein stability, and sustained PI3K/AKT and MAPK/ERK1/2 signaling. Benzyl-α-GalNAc reduced KIT O-GalNAcylation and stability, attenuated GALNT7-driven phenotypes, and inhibited xenograft growth.

**Conclusions:**

GALNT7-mediated O-GalNAc glycosylation stabilizes KIT and drives GIST progression. GALNT7 may serve as a prognostic biomarker and therapeutic target in GIST.

## Graphical Abstract

**Graphical Abstract:**
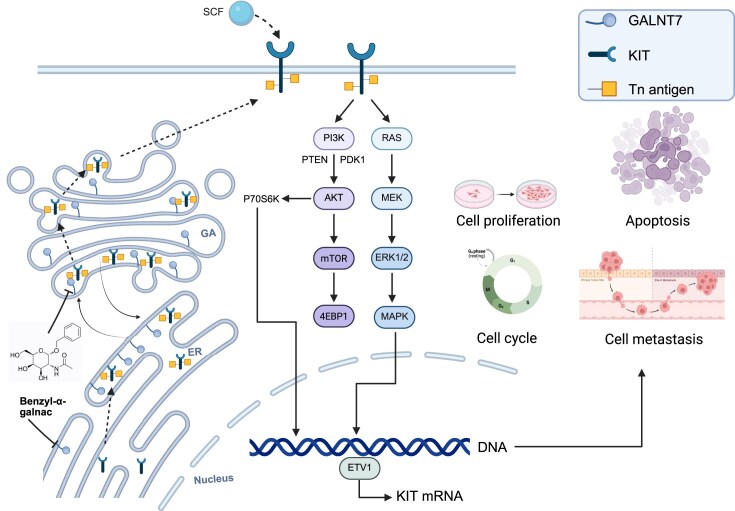
GALNT7-mediated O-GalNAc glycosylation stabilizes KIT, activates PI3K/AKT and MAPK/ERK signaling, and drives malignant progression of gastrointestinal stromal tumor (GIST); benzyl-α-GalNAc suppresses this axis.

## Introduction

Gastrointestinal stromal tumor (GIST) originates from precursor cells of the pacemaker interstitial cells of Cajal (ICC), which are the most common mesenchymal tumors of the gastrointestinal tract [1, 2]. Among all soft tissue sarcomas, GIST ranks first in incidence, with an annual rate of ~1–2 per 100 000 individuals. The stomach is the most frequent primary site (accounting for 60%–70% of cases), followed by the small intestine (20%–30%), while colorectal and esophageal locations are relatively rare. Activating mutations in the *c-KIT* gene are present in ~75%–80% of GIST cases, and 5%–10% harbor mutations in the platelet-derived growth factor receptor alpha (*PDGFRA*) gene [3]. Surgical resection is the cornerstone of curative therapy for localized GIST [4]. However, the high postoperative recurrence and metastasis rates, coupled with the limited efficacy of conventional radiotherapy and chemotherapy, contribute to an overall unfavorable prognosis for patients [5, 6]. Although tyrosine kinase inhibitors (TKIs) have substantially improved the treatment of advanced GIST, clinical progression remains a major challenge, highlighting the need to identify additional mechanisms that regulate KIT-driven oncogenic signaling and malignant progression [5, 7].

Protein glycosylation is a fundamental biochemical modification process wherein carbohydrate molecules form covalent bonds with functional groups on proteins [8, 9]. This ubiquitous process, occurring in eukaryotes, prokaryotes, and some viruses, is catalyzed by a diverse array of specific enzymes within intracellular compartments such as the Golgi apparatus, endoplasmic reticulum, or on the cell surface [10]. Based on the linkage between the glycan and the protein, glycosylation is primarily categorized into N-linked glycosylation, O-linked glycosylation, C-mannosylation, and glycosylphosphatidylinositol (GPI)-anchor attachment [11]. Among these, O-linked N-acetylgalactosamine (O-GalNAc) glycosylation represents one of the most prevalent forms of post-translational modification, affecting >80% of proteins traversing the Golgi apparatus, and is central to tissue development and essential for maintaining organismal homeostasis [12]. Notably, aberrant O-GalNAc glycosylation plays a key role in tumor progression. It not only generates tumor-associated carbohydrate antigens like the tumor-associated Tn antigen (GalNAc-α-O-Ser/Thr), which are specifically recognized by lectins such as Vicia villosa (VVA), but also serves as a hallmark of numerous human malignancies. It influences tumor progression by modulating diverse hallmarks of cancer, including malignant phenotype-related pathways and immune responses [13–15]. Current research indicates that the expression levels of certain tumor-associated carbohydrate antigens are significantly correlated with poor prognosis in various cancers [16, 17], spurring efforts to develop diagnostic reagents and vaccines targeting these antigens. However, the specific glycosylation-related genes involved in the synthesis of these antigens and their precise pathophysiological mechanisms in cancer remain incompletely understood [18]. Given the established close association between O-glycosylation and cancer, a thorough investigation into the specific regulatory mechanisms and functional roles of O-GalNAc glycosylation in GIST holds significant promise for unveiling novel pathogenic insights and devising innovative intervention strategies for GIST.

The O-GalNAc glycosylation process is mediated specifically by the polypeptide GalNAc-transferase (GALNT) family, which comprises 20 members. These enzymes mediate the covalent attachment of GalNAc from UDP-GalNAc to the hydroxyl group of serine or threonine residues on target proteins [12, 19]. Studies have demonstrated that GALNT family members exhibit dysregulated expression patterns in a wide range of malignancies, where they contribute to tumor progression via the GalNAc-type O-glycosylation of key proteins [20–23]. The expression of these enzymes is characterized by tissue specificity and spatiotemporal dynamics, with their isoform diversity increasing significantly alongside tissue complexity. This implicates GALNTs as key regulators in a spectrum of physiological and pathological processes by modulating intricate protein glycosylation networks. Accumulating evidence underscores the critical roles of GalNAc-type O-glycans and their corresponding GALNT genes in tumor biology. For instance, GALNT12 can activate the BMP signaling pathway by O-glycosylating the BMPR1A receptor, thereby inhibiting the bone-tropic metastatic capability of prostate cancer cells [24]. In pancreatic cancer, downregulation of GALNT3 alters the O-glycans on EGFR and HER2 receptors, consequently enhancing tumor cell invasiveness [25]. Furthermore, GALNT3 has emerged as a potential diagnostic biomarker for both lung and pancreatic cancers [26]. GALNT7 is known to drive prostate cancer growth by dysregulating global O-glycosylation. This functional role establishes it as a valuable dual-purpose target, serving both as a prognostic biomarker and a therapeutic avenue across multiple cancer types [17]. However, the precise mechanisms through which GALNT7 influences disease pathogenesis, including whether it acts via the specific glycosylation of key substrate proteins, remain largely unknown.

This study unveils a pivotal oncogenic role for GALNT7 in GIST. We experimentally demonstrated that GALNT7 overexpression significantly enhances the malignant phenotypes of GIST cells. Mechanistically, we discovered that GALNT7 mediates O-GalNAc glycosylation of the KIT receptor, thereby augmenting its protein stability and subsequently activating downstream oncogenic signaling pathways. Notably, we confirmed that the O-GalNAc glycosylation-specific inhibitor, Benzyl-α-GalNAc, effectively suppresses malignant progression in GIST. Our systematic investigation not only delineates a novel molecular pathway wherein GALNT7 regulates KIT protein stability via a distinct post-translational modification, but also nominates GALNT7 as a promising biomarker and therapeutic target in GIST.

## Methods

### Clinical data and tissue samples

This single-institution study was conducted at the Department of Gastrointestinal Surgery, The First Affiliated Hospital of Zhengzhou University. Archived formalin-fixed paraffin-embedded tissue blocks were collected from patients with pathologically confirmed GISTs who underwent curative surgical resection. Patients were included if they had a definite pathological diagnosis of GIST, available tumor and matched adjacent nontumor tissue blocks suitable for tissue microarray construction, and complete clinicopathological information. Patients were excluded if the pathological diagnosis was uncertain, the tissue blocks were unavailable or insufficient for tissue microarray preparation, the tissue quality was poor, or the essential clinical information was incomplete. Paired tumor and adjacent nontumor tissue samples were obtained from the eligible participants. This study was approved by the Institutional Ethics Committee of The First Affiliated Hospital of Zhengzhou University (approval no. 2024-KY-1592-001), and written informed consent was obtained from all participants in accordance with the Declaration of Helsinki.

### Cell culture

GIST-T1 cells were obtained from Cosmo Bio (Tokyo, Japan), and GIST-882 cells were kindly supplied by Professor Peng Zhang. Cell culture conditions are as described in the previous study [27]. For cryopreservation, cells were stored at −80°C in freezing vials (NEST Biotechnology) using CELLSAVING cryopreservation solution (New Cell & Molecular Biotech).

### Western blot

The method was performed as described previously [28]. Primary antibodies included anti-GALNT7 (1:2000, ab254971, Abcam), anti-KIT (1:2000, 18696-1-AP, Proteintech), biotinylated Vicia villosa agglutinin (VVA, 1:1000, B-1235, Vector), anti-β-actin (1:5000, 20536-1-AP, Proteintech), anti-p27, anti-p21, anti-cyclin A2, anti-cyclin B1 (Proteintech), anti-E-cadherin, anti-N-cadherin, anti-vimentin, anti-mTOR, anti-phospho-mTOR, anti-AKT, anti-phospho-AKT, anti-ERK1/2, anti-phospho-ERK1/2, and apoptosis antibody sampler kit (9915T, Cell Signaling Technology). Secondary antibodies were obtained from Proteintec.

### Co-immunoprecipitation

For co-immunoprecipitation (Co-IP) assays, cell lysates were prepared in RIPA lysis buffer with protease inhibitors. Lysates were incubated overnight at 4°C with anti-GALNT7 or anti-KIT. Protein A/G agarose beads were then added for an additional 2-hour incubation at 4°C. After washing the beads three times with cold lysis buffer, the immunoprecipitated complexes were eluted and analyzed by Western blotting. To assess KIT glycosylation, cell lysates were also subjected to lectin pull-down using streptavidin-agarose beads conjugated with biotinylated VVA, followed by immunoblotting for KIT.

### Liquid chromatograph–mass spectrometry analysis

GIST-T1 cells stably overexpressing Flag-tagged GALNT7 were lysed, and Flag-GALNT7-associated protein complexes were immunoprecipitated using an anti-Flag antibody coupled to Protein A/G beads. After quality inspection, the immunoprecipitated protein complexes were subjected to liquid chromatograph-mass spectrometry (LC–MS)/MS analysis using a Q Exactive HF-X mass spectrometer (Thermo Fisher Scientific). Raw MS data were processed using Proteome Discoverer software, version 3.1.0.638, with the Sequest HT search engine. The acquired MS/MS spectra were searched against the UniProt reviewed human protein database. Trypsin was selected as the digestion enzyme, allowing up to two missed cleavages. The precursor and fragment mass tolerances were set to 10 ppm and 0.02 Da, respectively. Carbamidomethylation of cysteine was set as a static modification, and oxidation of methionine was set as a dynamic modification. Peptide-spectrum matches were filtered using the Fixed Value PSM Validator with a maximum Delta Cn of 0.05.

### RT-qPCR

The extraction of total RNA, acquisition of CDNA, and detection of relative quantitative OCR were carried out in accordance with the instructions of Vazyme (Nanjing, China). Primer sequences are provided in supplementary Table 1. The data statistical method is as described in the previous study [27].

### Confocal laser scanning fluorescence microscopy

GIST-T1 cells grown on coverslips were fixed with cold methanol, permeabilized, and blocked with 5% normal goat serum. They were then incubated overnight at 4°C with primary antibodies against GALNT7 and KIT. Subsequently, different fluorescent secondary antibody labels were used to obtain the original protein co-localization images through confocal microscopy (Carl Zeiss, Germany).

### Cell viability assays and colony formation

The CCK8 reagent was purchased from APExBIO and tested according to the instructions. The colony formation assay as described in previous research [27].

### EDU assay

Cells were seeded in 24-well plates, treated as indicated, and then incubated with EdU labeling medium. After fixation and permeabilization, the incorporated EdU was detected via a click chemistry reaction, and nuclei were stained with Hoechst. Finally, images were acquired and analyzed using a fluorescence microscope.

### Cell transfection and lentiviral infection

Lentiviral vectors for GALNT7 knockdown (shGALNT7) and overexpression (OE-GALNT7) (NM_017423.3) were supplied by GeneChem (Shanghai, China). The targeting sequence for GALNT7 knockdown was 5′-GAGTCTATTAGAAGAATAAAC-3′. Lentiviral transduction was conducted according to the protocol [27], and stable cell lines were selected and maintained in medium containing 4 µg/ml puromycin.

### Wound scratch assay

Cells were seeded in six-well plates. A uniform wound was created across the cells using a sterile pipette tip. After washing to remove debris, cells were cultured in serum-free medium. Cell migration ability was assessed by comparing phase-contrast microscope images taken at 0 and 24 hours at six predetermined points along the scratch wound, quantifying the wound closure capacity.

### Cell migration and invasion analysis

The migration and invasion assays were evaluated using Transwell (8.0 μm, Cat. #3422; Corning Inc., NY, USA). The method was performed as described previously [24].

### Cell cycle and apoptosis

Cells (~1 × 10^6^) were harvested and washed twice with PBS. Subsequently, cold 70% ethanol was added dropwise to the cell pellet under gentle vortexing, followed by fixation at 4°C for at least 4 hours. After fixation, removed ethanol, washed, and resuspended in PBS. Cell permeabilization was performed using PBS containing 0.1% Triton X-100, followed by incubation with a staining solution containing 50 μg/ml PI and 100 μg/ml RNase A at 37°C for 0.5 hours in the dark, subsequently detected by flow cytometry. Apoptosis was detected using an Annexin V-FITC/PI double staining kit. Cells were stained according to the instructions and analyzed by flow cytometry.

### Immunohistochemistry and immunofluorescence

IHC staining as described in previous study [28]. For IF staining on cells or frozen tissue sections, samples were fixed, blocked, and incubated with primary antibodies overnight, followed by fluorophore-conjugated secondary antibodies [27]. Slides were imaged using a confocal microscope (Nikon C1s, Japan), and fluorescence intensity was analyzed with ImageJ software.

### Hematoxylin and eosin staining

To examine pathological changes in mouse liver tissue, paraffin-embedded sections were deparaffinized in xylene and rehydrated through a graded ethanol series. The sections were stained with hematoxylin at room temperature for 5 min, followed by immersion in water for 2 min to remove excess dye. Subsequently, the sections were differentiated in differentiation solution (1% hydrochloric acid in ethanol) for several seconds, up to 30 s. After differentiation, the sections were rinsed in water for 30–60 min, counterstained with eosin for 2 min, and finally rinsed with water for further analysis.

### Xenograft nude mouse model

Four-week-old female BALB/c mice were subcutaneously inoculated in the right anterior axilla with stable GALNT7-knockdown GIST-T1 cells (5.0 × 10⁶ cells) for the experimental group (*n* = 6) or control cells for the control group (*n* = 6). In a separate experiment, mice were inoculated with GIST-T1 cells (5.0 × 10^6^ cells) and assigned to either a control group (*n* = 5) or a group treated with the Benzyl-α-GalNAc inhibitor (*n* = 5). All mice were euthanized 24 days postinoculation. Throughout the experimental period, weight and tumor size were measured every 4 days. Tumor volume was calculated using the formula: 1/2 × (length × width²). All animal experiments in this study were approved by the Institutional Animal Care and Use Committee of Henan Institute of Medical and Pharmaceutical Sciences (approval No. 2025-YYY-052).

### Liver metastasis model

Liver metastases were induced in 8- to 10-week-old BALB/c-nu mice by intrasplenic injection of 3  ×  10⁵ GIST-T1 cells (control and GALNT7-knockdown groups). An additional group received 60 μl of PBS alone or PBS mixed with Benzyl-GalNAc. Four weeks after injection, four mice randomly selected from each group were euthanized for liver collection, macroscopic imaging, and hematoxylin-eosin staining. The remaining mice (*n* = 6 per group) were monitored for 2 months to evaluate survival rates.

### Bulk RNA sequencing data preprocessing and analysis

Publicly available GIST RNA-seq data (HRA005970) were downloaded from the NGDC database. SRA files were converted to FASTQ format using fastq-dump (v3.0.6) and assessed for quality using FastQC (v0.11.9); samples failing “Per sequence quality scores” or “Per base sequence quality” metrics were excluded. Low-quality reads were filtered using fastp (v0.23.2) with default parameters. The resulting clean reads were aligned to the human reference genome (GRCh38) using HISAT2 (v2.2.1). Transcript abundance was quantified via featureCounts (v2.0.3). Finally, we performed variance stabilizing transformation (vst) normalization and differential expression analysis using DESeq2 (v1.36.0).

### Weighted gene co-expression network analysis

We adopted the weighted gene co-expression network analysis (WGCNA) (v1.73) [29] to identify significant gene modules. Initially, Pearson correlation coefficients were calculated for all genes across the samples filtered via hierarchical clustering. These correlations were transformed into an adjacency matrix using a soft-thresholding power of *β* = 6. Subsequently, the topological overlap matrix (TOM) was calculated using the TOM similarity function, followed by hierarchical clustering to generate a gene dendrogram. Modules were defined using the dynamic tree-cutting algorithm, which groups genes with similar expression patterns into distinct, color-coded clusters. Finally, modules were filtered based on module-trait correlations (threshold: |*r*| > 0.5 and *P* < 0.05) to select candidates for downstream analysis.

### Single-cell RNA sequencing data preprocessing and analysis

The scRNA-seq data were obtained from GSE254762. Preprocessing was conducted using R package Seurat (v5.1.0) [30]. The data underwent rigorous quality control filtering. Cells were retained only if they met the following criteria: 500–6000 nFeature_RNA, <40 000 nCount_RNA, and <15% mitochondrial gene expression. Doublet removal was conducted using Scrublet [31], with a doublet rate threshold of 0.06 and a score threshold of 0.25. Following filtration, the expression matrix was normalized using the LogNormalize methods (scale factor: 10 000). PCA was performed using the top 2000 highly variable genes identified via FindVariableFeatures. Initial cell clustering was conducted using the FindClusters function at a resolution of 0.6. For subsequent subcluster analysis, we optimized clustering resolutions based on visual inspection. To determine the markers for each cluster, we utilized the FindAllMarkers and FindMarkers functions within Seurat.

### Calculation of the signature score

For the scRNA-seq data, we calculated individual cell scores for the gene signature using the AddModuleScore function within Seurat. This method calculates the average expression of the target gene set while correcting for the aggregated expression of control gene sets. For bulk data analysis, we evaluated gene signature scores using ssGSEA by GSVA (v2.0.5) package [32].

### Inferring single-cell copy-number variation

Copy-number variations (CNVs) were estimated using the R package infercnv (v1.22.0) [33]. To avoid batch effects, the analysis was performed individually for each sample. Fibroblasts, SMCs, and ECs were included as the query populations, while T cells were selected as the nonmalignant reference group. The analysis was conducted using the following parameters: cutoff = 0.1, denoise = TRUE, and HMM = FALSE. Chromosomes Y and M were excluded from the analysis.

To quantify the CNV levels, the inferCNV expression values were first Z-score standardized and then linearly scaled to strictly fall within the range of [−2, 2]. For each cell, the CNV score was calculated as the mean of the squared scaled values across the genome. Additionally, to assess the similarity to a malignant profile, we identified the top 5% of cells with the highest CNV scores to generate a consensus high-CNV profile by averaging their scaled expression. The CNV correlation for each cell was then determined by calculating the Pearson correlation coefficient between its CNV profile and this high-CNV consensus profile.

### Trajectory analysis

Cell differentiation trajectories were constructed using Monocle2 (v2.34.0) [34], employing the default settings suggested by the developers. To identify potential differentiation start points, we employed the R package CytoTRACE (v0.3.3) to predict cellular developmental potential [35].

### Statistical analysis

We use GraphPad Prism 7.0 to perform statistical analysis on the experimental results, with each result repeated at least 3 times independently. The data were expressed as the mean ± SEM. *P*-values <.05 are considered meaningful.

## Results

### Multiomics data identify significant activation of O-glycosylation in GIST

**Figure 1:**
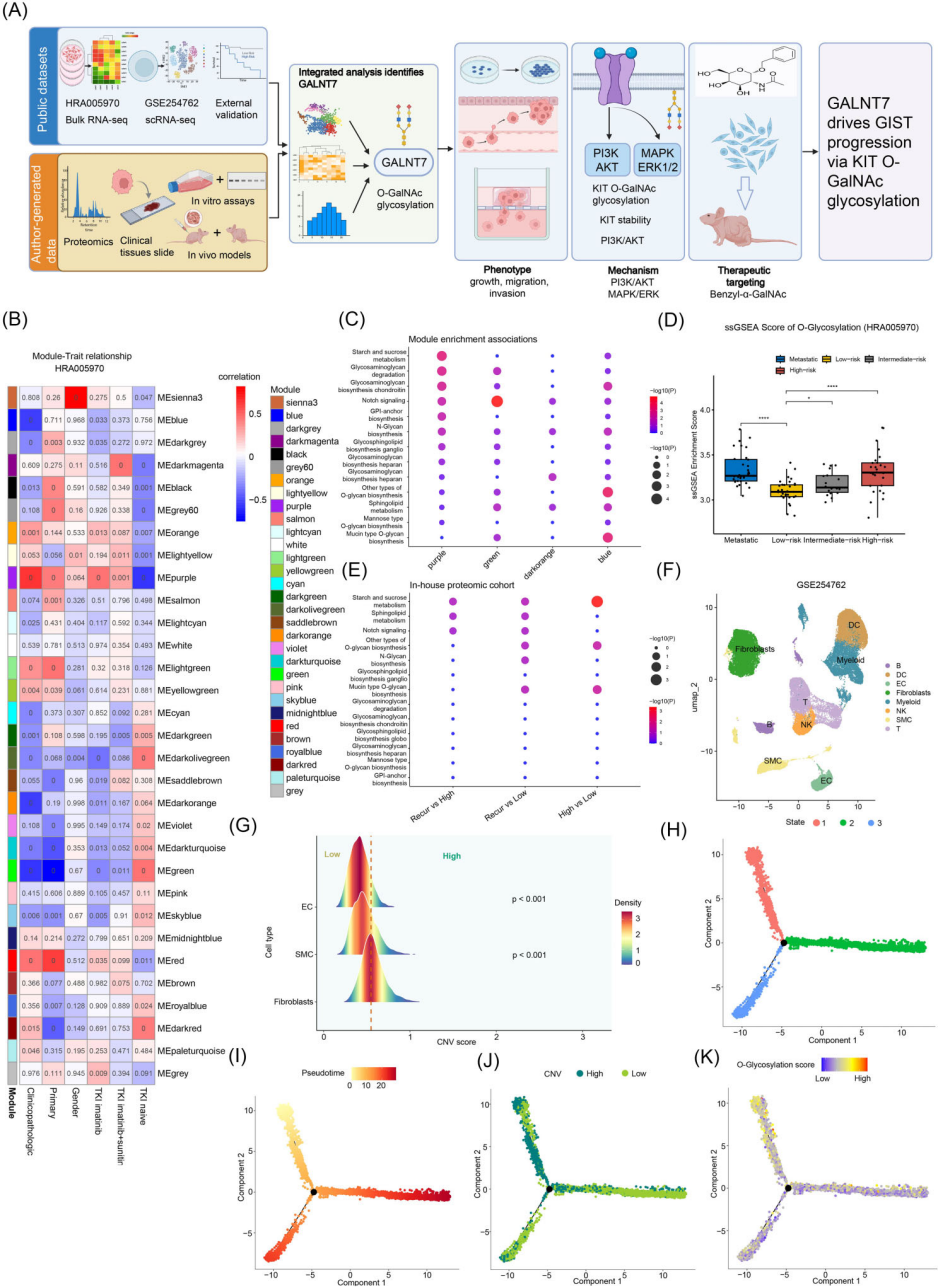
Multiomics analyses reveal significant activation of O-glycosylation in GIST. (**A**) Schematic overview of the study design, showing public dataset analyses and author-generated experiments. (**B**) Heatmap showing correlations between WGCNA-derived gene modules and clinicopathological risk grades in 113 GIST samples from the HRA005970 bulk RNA-seq cohort. Modules significantly associated with pathological risk were selected using the criteria |*R*| > 0.5 and *P* < 0.05. (**C**) Functional enrichment analysis of genes from the purple, green, dark orange, and blue modules identified in WGCNA. Dot plots show representative biological processes enriched in each module, highlighting O-glycosylation-related pathways. (**D**) ssGSEA-based O-glycosylation signature scores in GIST samples stratified by pathological risk grade. Higher scores indicate increased activation of O-glycosylation-related transcriptional programs. (**E**) Functional enrichment analysis of differentially expressed proteins identified from in-house proteomic profiling of 12 GIST samples. Comparisons included high-risk versus low-risk, recurrent versus high-risk, and recurrent versus low-risk groups, with enrichment of O-glycosylation-related processes observed across risk-associated comparisons. (**F**) UMAP visualization of 53 201 single cells from nine GIST samples in the GSE254762 dataset after quality control and clustering. Major cell populations were annotated according to canonical marker genes, including fibroblasts, dendritic cells, T cells, myeloid cells, NK cells, smooth muscle cells, endothelial cells, and B cells. (**G**) Ridgeline plot showing the distribution of inferred CNV scores among stromal cell populations. Fibroblasts displayed higher CNV levels than smooth muscle cells and endothelial cells, suggesting that high-CNV fibroblast-like cells may represent malignant GIST cells. The red dashed line indicates the threshold used to stratify cells into high- and low-CNV groups. (**H–K**) Monocle2-based pseudotime trajectory analysis of fibroblast-like cells. Cells are colored according to cell state (**H**), pseudotime ordering (**I**), CNV level (**J**), and O-glycosylation signature score (**K**). High O-glycosylation scores were enriched in early pseudotime states with elevated CNV levels, suggesting that O-glycosylation activation is associated with malignant cellular states in GIST. *P < 0.05, **P < 0.01, ***P < 0.001, ****P < 0.0001.

The overall experimental design of this study is summarized in Fig. 1A, which distinguishes analyses based on publicly available datasets from experiments performed by the authors. To investigate the extent of O-glycosylation activation in GIST, we first collected RNA-seq data including 113 patients from HRA005970 and performed WGCNA. Stringent thresholds (|*R*| > 0.5 and *P* < 0.05) were applied to pinpoint critical modules related to GIST pathological risk grades, resulting in the identification of four critical modules (Fig. 1A). Functional enrichment analysis revealed that these modules were significantly enriched in O-glycosylation-related biological processes (Fig. 1B). Moreover, scoring of these samples using the ssGSEA algorithm for O-glycosylation signature demonstrated significant differences in the score across different pathological risk grades (Fig. 1C). Additionally, we performed sequencing in proteomics for 12 in-house GIST samples and carried out enrichment analysis of differentially expressed proteins across pathological grades. The results showed significant enrichment of O-glycosylation-related biological processes (Fig. 1D). These findings suggest a potential association between GIST pathological grading and O-glycosylation.

To delve deeper into the relationship between GIST and O-glycosylation, single-cell sequencing data from nine GIST cases (GSE254762) was analyzed. After quality control, we obtained transcriptomic profiles for 53 201 cells, which were grouped into 23 clusters via UMAP visualization (supplementary Fig. 1A). These cells were annotated into distinct cell types, including fibroblasts, dendritic cells (DC), T cells, myeloid cells, natural killer (NK) cells, smooth muscle cells (SMC), endothelial cells (EC), and B cells (Fig. 1E) and marker gene expression to confirm the reliability of our annotations (supplementary Fig. 1B). Notably, the stromal cells exhibited significant heterogeneity, comprising fibroblasts (14 936, 67.11%), SMC (4270, 19.19%), and EC (3050, 13.7%), and these cell types have now been implicated in GIST tumorigenesis [36–39]. To accurately characterize the tumor cell landscape in GIST, the three cell types were subjected to inferCNV analysis. While SMC and EC showed limited CNVs, fibroblasts displayed substantial alterations (supplementary Fig. 2A). Based on the inferred single-cell CNV spectrum, we found that fibroblasts displayed significantly elevated CNV levels compared to SMC and EC (Fig. 1F and supplementary Fig. 2B–D). Subsequently, CopyKAT algorithm was employed to calculate chromosome ploidy and distinguish tumor populations. SMC and EC displayed normal diploid profile (supplementary Fig. 3). We hypothesize that fibroblasts with high CNV levels are likely tumor cells, and fibroblasts were retained for downstream investigation consequently. The fibroblasts were stratified into low and high groups according to their CNV levels (Fig. 1F and supplementary Fig. 2D). To comprehensively investigate the role of O-glycosylation on the evolutionary dynamics of fibroblasts within GIST, Monocle2 and cytoTRACE algorithm were conducted for unsupervised trajectory analysis. As a result, both of which indicated a comparable evolutionary path, with fibroblasts stemming from more malignant cells (Fig. 1G–I and supplementary Fig. 4). Importantly, high O-glycosylation scores were predominantly localized in the initial differentiation state S1, whereas the low scores were concentrated in S2 and S3 (Fig. 1J). The data suggested that enhanced O-glycosylation activity and high CNV levels appeared essential to sustain the malignant characteristics of GIST. Overall, these findings demonstrate that O-glycosylation is significantly activated in GIST.

### Elevated GALNT7 is associated with O-glycosylation in GIST

**Figure 2:**
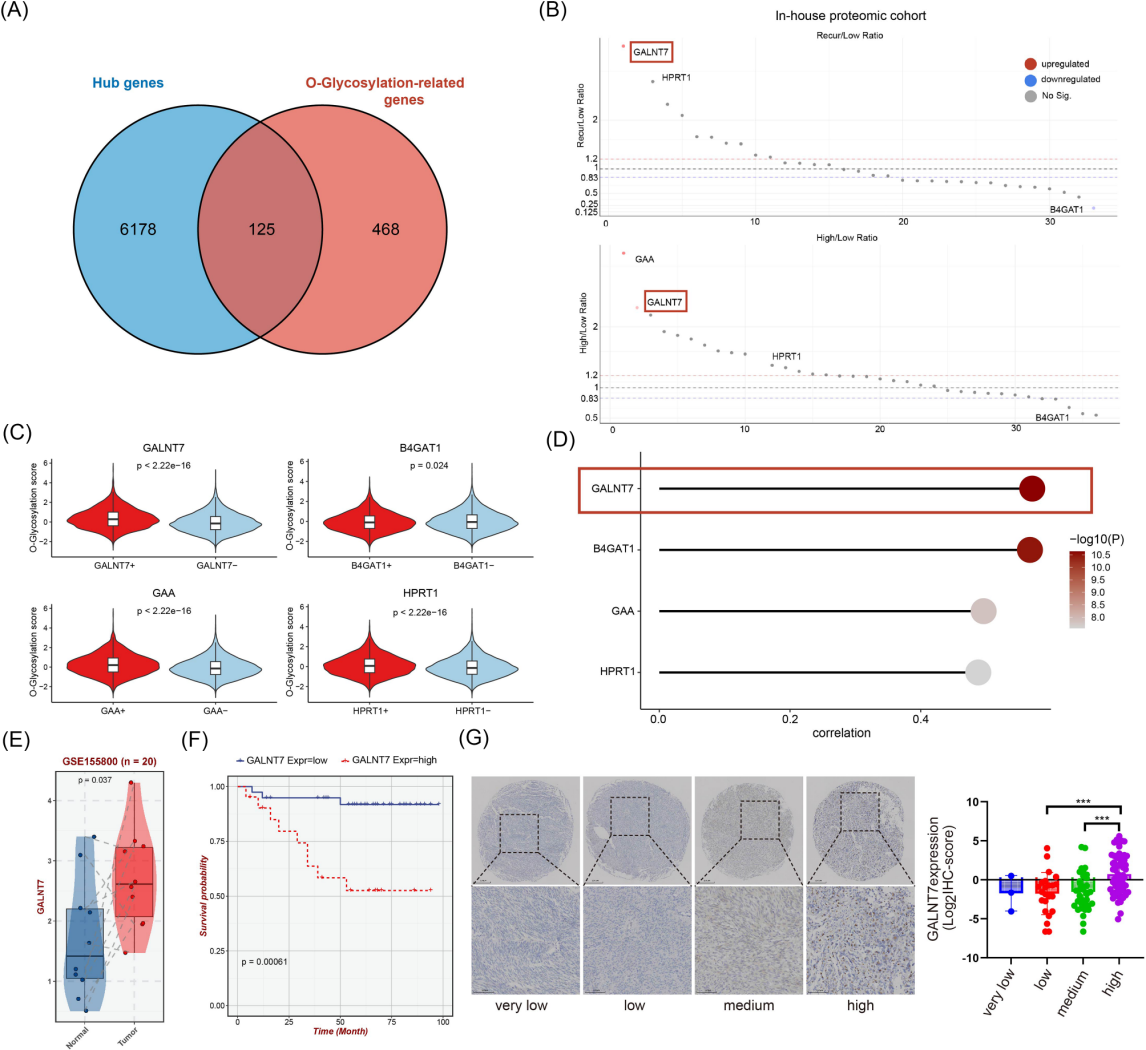
GALNT7 is identified as a hub factor associated with O-glycosylation activation in GIST. (**A**) Venn diagram showing the intersection between hub genes from the four risk-associated WGCNA modules and O-glycosylation-related genes, resulting in 125 candidate genes potentially involved in O-glycosylation activation in GIST. (**B**) Scatter plot showing differentially expressed proteins from the in-house proteomic dataset. Among the candidate genes, *GALNT7, GAA*, and *HPRT1* were significantly upregulated, whereas *B4GAT1* was significantly downregulated. (**C**) Violin plots comparing scaled O-glycosylation signature scores between gene-positive and gene-negative fibroblast-like cells for *GALNT7, B4GAT1, GAA*, and *HPRT1. GALNT7*-positive cells exhibited markedly higher O-glycosylation scores than *GALNT7*-negative cells. Statistical significance was assessed using the Wilcoxon rank-sum test. (**D**) Lollipop plot showing correlations between the expression of *GALNT7, B4GAT1, GAA*, and *HPRT1* and their corresponding module eigengenes. GALNT7 showed the strongest association with the risk-related O-glycosylation module. (**E**) Box plot showing *GALNT7* expression levels in GIST and normal tissues using the GSE155800 dataset obtained from dbGIST. (**F**) Kaplan–Meier analysis showing progression-free survival of patients with GIST stratified by GALNT7 expression. Patients with high GALNT7 expression exhibited significantly shorter progression-free survival. (**G**) Representative immunohistochemical staining images of GALNT7 in low-risk and high-risk GIST tissues. GALNT7 staining intensity was increased in high-risk GIST samples. Scale bar = 50 μm. *P < 0.05, **P < 0.01, ***P < 0.001, ****P < 0.0001.

To identify the key factors underlying O-glycosylation activation in GIST, we intersected hub genes from the four critical WGCNA modules (blue, purple, green, and dark orange) with genes associated with O-glycosylation, yielding 125 candidates (Fig. 2A). Among these, GALNT7, GAA, and HPRT1 were significantly upregulated, while B4GAT1 was significantly downregulated in the in-house proteomic dataset (Fig. 2B). Furthermore, GALNT7+, GAA+, and HPRT1+ fibroblasts exhibited higher O-glycosylation scores compared with the negative counterparts (Fig. 2C), with *GALNT7* showing the strongest correlation with corresponding module eigengenes (Fig. 2D). These findings suggest that *GALNT7* is the most likely hub gene driving O-glycosylation activation in GIST. External datasets from dbGIST (https://www.dbgist.com/) further demonstrated that upregulated GALNT7 was associated with poor progression-free survival in GIST (Fig. 2E and F). Consistently, immunohistochemical analysis demonstrated that GALNT7 expression was significantly elevated in high-risk GIST tissues compared with low- and intermediate-risk samples (Fig. 2G). To further assess the clinical relevance of GALNT7, we examined its association with the clinicopathological characteristics of 120 patients with GIST. The results revealed that GALNT7 expression was significantly correlated with age, recurrence status, and administration of adjuvant therapy (supplementary Table 2). Collectively, these results indicate that upregulated *GALNT7* functions as a hub gene and is closely associated with aberrant O-glycosylation activation in GIST.

### GALNT7 regulates the proliferation of GIST cells both *in vitro* and *in vivo*

**Figure 3:**
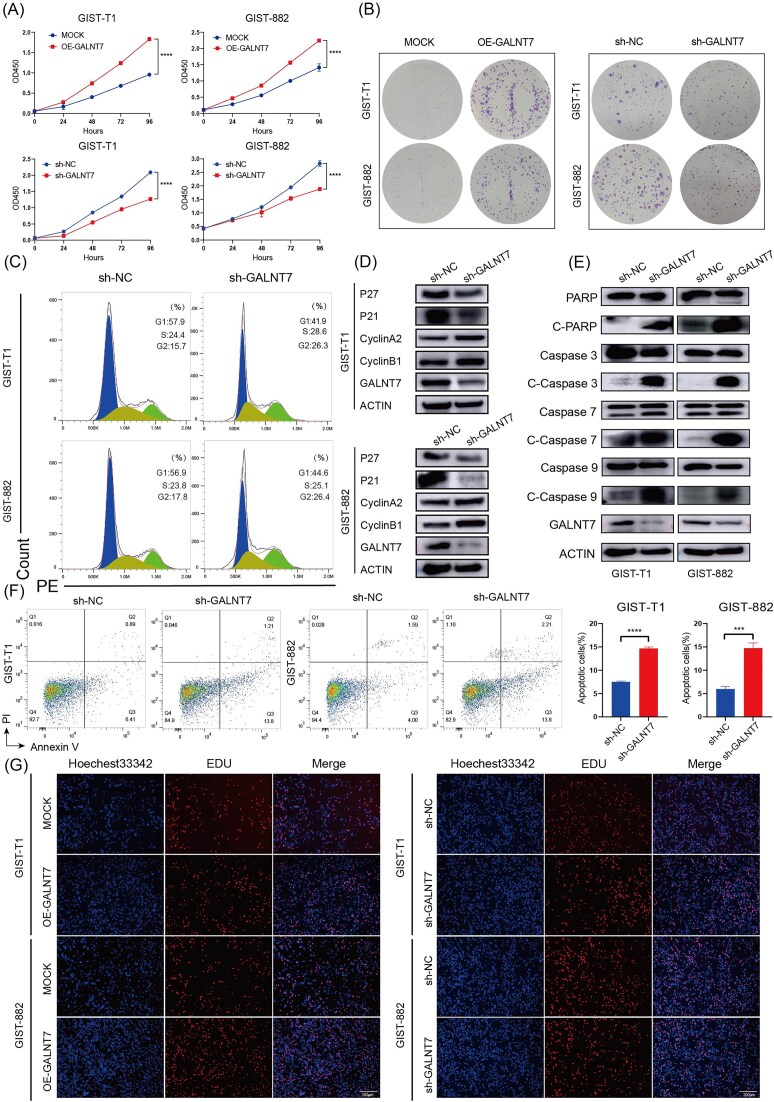
GALNT7 promotes the proliferation and survival of GIST cells *in vitro*. (**A**) CCK-8 assays showing the effects of *GALNT7* knockdown or overexpression on the proliferation of GIST-T1 and GIST-882 cells. (**B**) Colony formation assays evaluating the long-term proliferative capacity of GALNT7-modulated GIST cells. (**C**) Flow cytometric analysis of cell cycle distribution in GIST cells after *GALNT7* knockdown or overexpression. (**D**) Western blot analysis of cell cycle-related proteins after *GALNT7* knockdown. (**E**) Western blot analysis of apoptosis-related proteins after *GALNT7* knockdown, showing activation of apoptotic signaling. (**F**) Annexin V-FITC/PI flow cytometry analysis assessing apoptosis in GALNT7-modulated GIST cells. (**G**) EDU incorporation assay showing DNA synthesis and proliferative activity in GIST cells after *GALNT7* knockdown or overexpression. OV-GALNT7, GALNT7 overexpression lentivirus; Mock, control lentivirus; shGALNT7, GALNT7 shRNA lentivirus; shNC, negative control shRNA lentivirus. Data are presented as mean ± SEM from at least three independent experiments. *P < 0.05, **P < 0.01, ***P < 0.001, ****P < 0.0001.

To explore the role of GALNT7 in the proliferation of GIST cells, we established stable *GALNT7*-knockdown and overexpressing clones in GIST-T1 and GIST-882 cells. Efficient modulation of GALNT7 expression was confirmed by Western blot and RT-qPCR analyses (supplementary Fig. 5A and B). CCK-8 assays revealed that *GALNT7* knockdown significantly suppressed cell growth, whereas its overexpression promoted proliferation in both cell lines (Fig. 3A). Similarly, colony formation and EDU assay consistently demonstrated that GALNT7 deficiency inhibited proliferative capacity, while its overexpression enhanced it (Fig. 3B and G and supplementary Fig. 5C and D). Flow cytometric analysis further indicated that altered GALNT7 expression disturbed cell cycle progression and promoted apoptosis (Fig. 3C–F and supplementary Fig. 5E).

**Figure 4:**
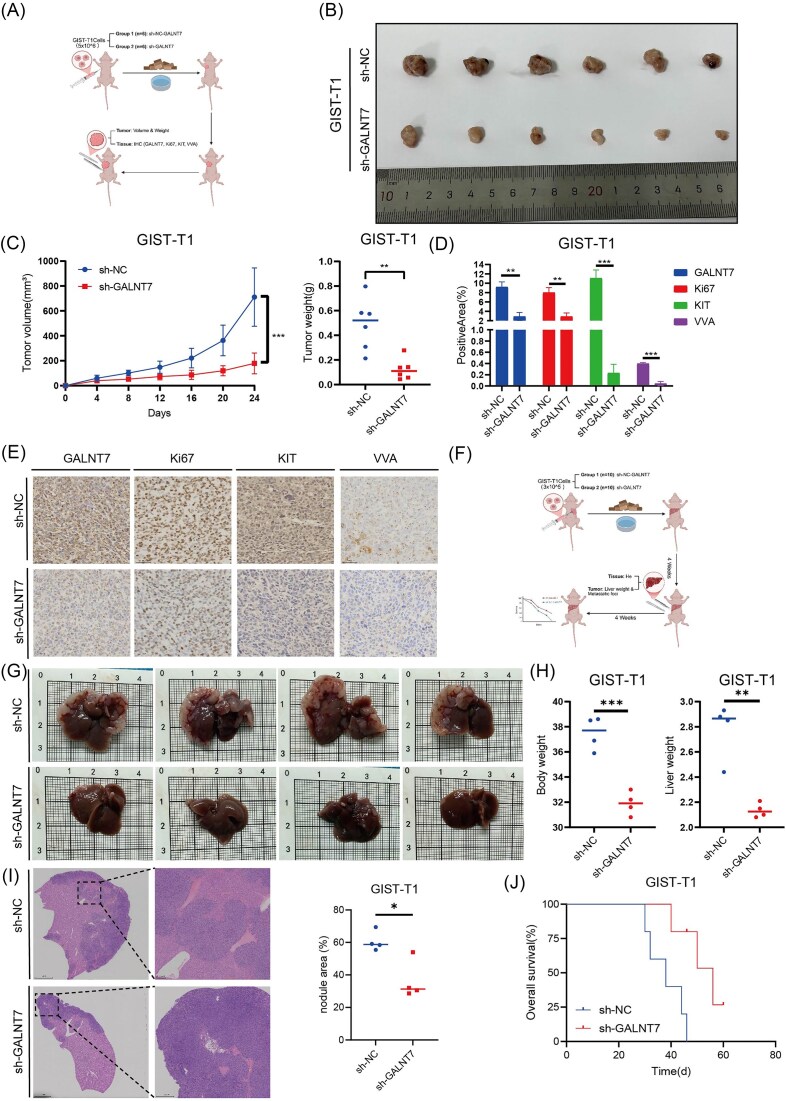
*GALNT7* knockdown suppresses GIST tumor growth and liver metastasis *in vivo*. (**A**) Schematic of the xenograft nude mouse experimental procedure. (**B**) Representative images of subcutaneous xenograft tumors derived from shNC and shGALNT7 GIST-T1 cells. (**C**) Tumor growth curves showing that *GALNT7* knockdown significantly reduced xenograft tumor volume over time. (**D**) Quantification of tumor weights at the endpoint, demonstrating decreased tumor burden in the shGALNT7 group compared with the shNC group. (**E**) Ki67, GALNT7, KIT, and VVA staining of tumor tissues (bar = 50 µm). (**F**) Hepatic metastasis nude mouse experimental flowchart. (**G**) Representative macroscopic images of livers from mice bearing metastatic tumors in the shNC and shGALNT7 groups. (**H**) Quantification of mouse body weight and liver weight in the liver metastasis model. (**I**) H&E-stained liver metastases and lesional regions derived from GIST-T1 cell. Scale bar, 250 μm. (**J**) Kaplan–Meier survival curve for tumor-bearing mice (*n* = 6). shGALNT7, GALNT7 shRNA lentivirus; shNC, negative control shRNA lentivirus. Data are presented as mean ± SEM. *P < 0.05, **P < 0.01, ***P < 0.001, ****P < 0.0001.

To extend these findings *in vivo*, a subcutaneous xenograft model was established (Fig. 4A). Compared with the control group, mice injected with shGALNT7-transduced cells exhibited significantly smaller tumor volumes and lower tumor weights (Fig. 4B and C), indicating that *GALNT7* knockdown effectively suppresses tumor growth *in vivo*. Immunohistochemical analysis of xenograft tissues showed decreased expression of KIT and Ki67 in the GALNT7-knockdown group, further supporting a role for GALNT7 in promoting GIST proliferation. Representative images also revealed concordant changes in GALNT7 and VVA staining patterns (Fig. 4D and E).

### GALNT7 regulates the migration and invasion of GIST cells both *in vitro* and *in vivo*

Given the poor prognosis of GIST patients with hepatic metastasis [40, 41], we hypothesized that GALNT7 influences metastatic potential by modulating key phenotypic features of metastasis, specifically cell migration and invasion *in vitro*. Wound healing assays demonstrated that GALNT7 overexpression enhanced GIST cell migration, whereas its knockdown suppressed this process (supplementary Fig. 6A and B). Transwell migration and invasion assays further revealed that GALNT7 overexpression promoted the migratory and invasive capacities of GIST-T1 and GIST-882 cells, while *GALNT7* knockdown significantly attenuated these abilities (supplementary Fig. 6C and D). Western blot and immunofluorescence analyses showed that modulation of GALNT7 expression led to marked alterations in the expression of metastasis-related markers (supplementary Fig. 6E–G), suggesting a promotive role of GALNT7 in GIST cell metastasis. To evaluate the role of GALNT7 in tumor progression *in vivo*, the researchers established a liver metastasis animal model (Fig. 4F). In this model, suppression of GALNT7 expression resulted in significantly lower body weight of nude mice and reduced mass of the lesioned livers compared to the control group (Fig. 4G–I). Furthermore, it markedly decreased both the number and size of hepatic metastatic lesions and led to a significant prolongation of overall survival in the animals. Additionally, an inverse correlation was found between GALNT7 expression and survival time in tumor-bearing animals (Fig. 4J). Collectively, these findings indicate that GALNT7 plays an important role in enhancing the invasive and metastatic potential of GIST.

### GALNT7 regulates the PI3K/AKT and MAPK/ERK signaling pathways in GIST by modulating KIT protein stability

**Figure 5:**
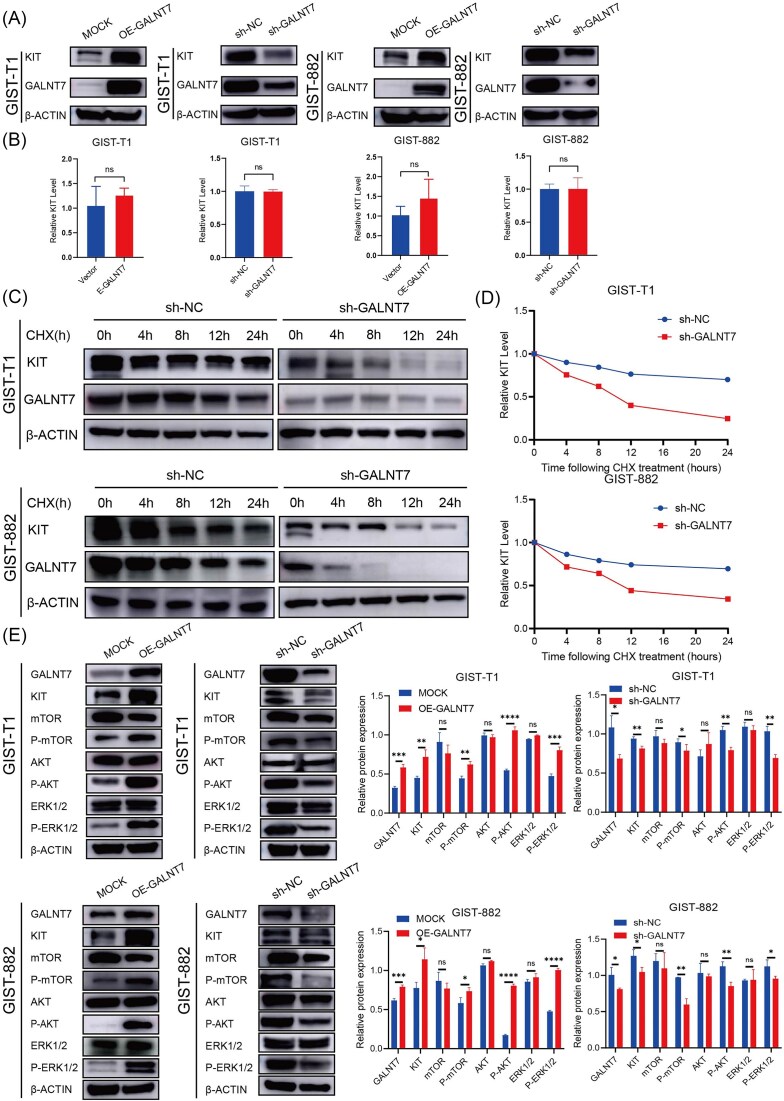
GALNT7 regulates the PI3K/AKT and MAPK/ERK signaling pathways in GIST by modulating KIT protein stability. (**A**) Western blot analysis showing KIT protein levels in GIST-T1 and GIST-882 cells after *GALNT7* knockdown or overexpression. *GALNT7* knockdown decreased KIT protein expression, whereas *GALNT7* overexpression increased KIT protein levels. (**B**) RT-qPCR analysis showing *KIT* mRNA expression after *GALNT7* knockdown or overexpression. (**C**, **D**) After cycloheximide (CHX) treatment, the half-life of the endogenous KIT protein, which had been lowered by *GALNT7* knockdown, was examined. (**E**) Effects of *GALNT7* overexpression and knockdown on the PI3K/AKT and MAPK/ERK signaling pathways.

Accumulating evidence underscores the critical role of *KIT* mutations in GIST [42]. Our immunohistochemical analysis of xenograft tissues revealed a concordant expression pattern between GALNT7 and KIT, prompting us to investigate whether GALNT7 regulates KIT expression. Western blot and RT-qPCR analyses demonstrated that modulation of GALNT7 expression influenced KIT protein levels but did not significantly alter its mRNA expression (Fig. 5A and B), suggesting post-transcriptional regulation. As GALNT7 is a glycosyltransferase and O-glycosylation is known to regulate protein stability, we examined its effect on KIT protein. Cycloheximide chase assays showed that *GALNT7* overexpression significantly prolonged the half-life of KIT, whereas *GALNT7* knockdown accelerated its degradation (Fig. 5C and D).

GIST pathogenesis is primarily driven by oncogenic KIT mutations, which constitutively activate multiple downstream signaling pathways, including PI3K/AKT and MAPK/ERK, thereby promoting tumor cell proliferation, survival, and resistance to apoptosis [43–45]. To determine whether GALNT7 modulates these pathways, we assessed the phosphorylation levels of key signaling molecules. We found that *GALNT7* knockdown markedly reduced the phosphorylation of AKT and ERK1/2, while its overexpression enhanced their activation (Fig. 5E).

Taken together, these results indicate that GALNT7 regulates the stability of the KIT protein, thereby modulating the activity of the PI3K/AKT and MAPK/ERK signaling pathways in GIST.

### GALNT7 promotes GIST tumorigenesis by regulating O-GalNAc glycosylation of KIT

**Figure 6:**
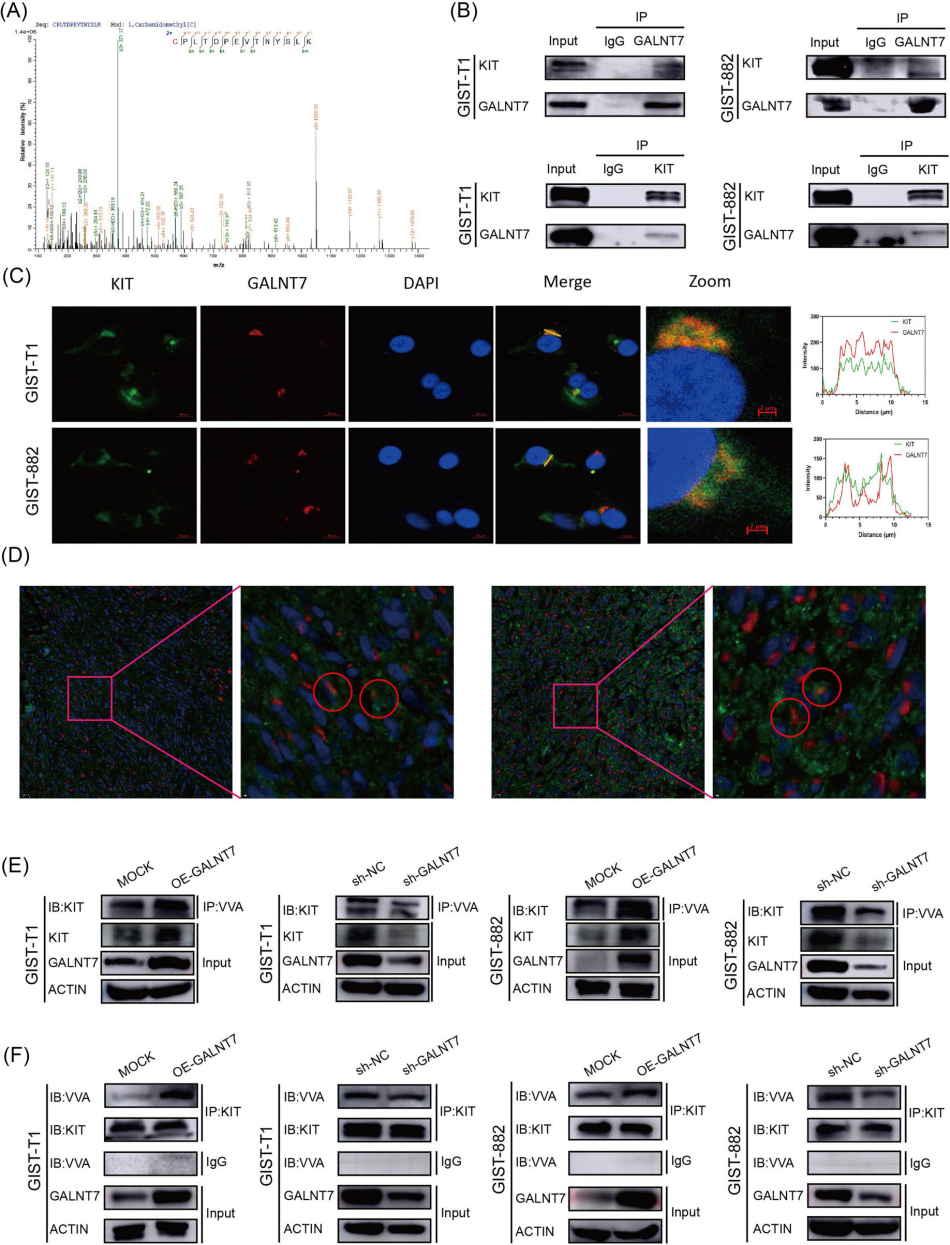
GALNT7 promotes GIST tumorigenesis by regulating O-GalNAc glycosylation of KIT. (**A**) LC–MS/MS analysis of proteins Co-IP with Flag-tagged GALNT7 in GIST-T1 cells. KIT was identified as a candidate GALNT7-interacting protein. (**B**) Reciprocal Co-IP assays confirming the physical interaction between endogenous GALNT7 and KIT in GIST-T1 and GIST-882 cells. Cell lysates were immunoprecipitated with anti-GALNT7 or anti-KIT antibodies and immunoblotted for the reciprocal protein. (**C**) Immunofluorescence staining showing intracellular colocalization of GALNT7 and KIT in GIST-T1 cells. GALNT7 was labeled in red, KIT in green, and nuclei were counterstained with DAPI. Merged images indicate spatial overlap between GALNT7 and KIT. (**D**) Dual immunofluorescence staining of primary GIST tissues showing colocalization of GALNT7 and KIT in tumor cells, supporting the GALNT7–KIT interaction in clinical samples. (**E, F**) GALNT7 modification of O-glycosylation of KIT measured by Co-IP in GIST cells. Cell lysates were immunoprecipitated (IP) for KIT, and then western blotted with biotin-conjugated VVA to distinguish Tn antigen expression. Lectin pull down glycoproteins using biotinylated VVA, and streptavidin agarose immunoblotted with anti-KIT antibody.

To elucidate the downstream mechanisms of GALNT7, we performed mass spectrometry-based proteomic screening. Proteins Co-IP with GALNT7 in GIST-T1 cells were analyzed by LC–MS/MS. After excluding proteins enriched in the IgG control group, KIT ranked among the top 30 candidates based on the Score Sequest HT value, supporting KIT as a prominent candidate in the GALNT7 interactome (Fig. 6A). Co-IP assays using both anti-GALNT7 and anti-KIT antibodies confirmed a physical interaction between GALNT7 and KIT in GIST-T1 cell (Fig. 6B). Furthermore, immunofluorescence analysis demonstrated substantial spatial colocalization of GALNT7 (red fluorescence) and KIT (green fluorescence) in GIST-T1 cells (Fig. 6C). In primary GIST patient tissue samples, immunofluorescence double staining demonstrated significant co-localization of GALNT7 protein (red fluorescence signal) and KIT receptor (green fluorescence signal) at the plasma membrane and intracellular regions (Fig. 6D). This finding corroborates our *in vitro* experimental results and further supports the molecular mechanism by which GALNT7 directly binds to KIT to regulate its glycosylation modification.

To investigate whether GALNT7 modulates O-glycosylation of KIT, we used VVA, which recognizes tumor-associated Tn antigens. Lysates from GIST cells with *GALNT7* knockdown or overexpression were immunoprecipitated with an anti-KIT antibody and blotted with biotinylated VVA. *GALNT7* overexpression enhanced VVA binding to KIT in both GIST-T1 and GIST-882 cells, whereas *GALNT7* knockdown reduced it (Fig. 6E). Consistent with these findings, streptavidin pulldown assays using biotinylated VVA followed by immunoblotting with an anti-KIT antibody showed that *GALNT7* overexpression increased KIT levels in VVA-precipitated glycoproteins, while *GALNT7* knockdown decreased them (Fig. 6F).

Collectively, these results demonstrate that GALNT7 interacts with KIT, promoting its O-GalNAcylation and thereby enhancing KIT protein stability, as well as activating downstream signaling pathways.

### Targeting the GALNT7–KIT axis with benzyl-α-GalNAc attenuates malignant progression in GIST

**Figure 7:**
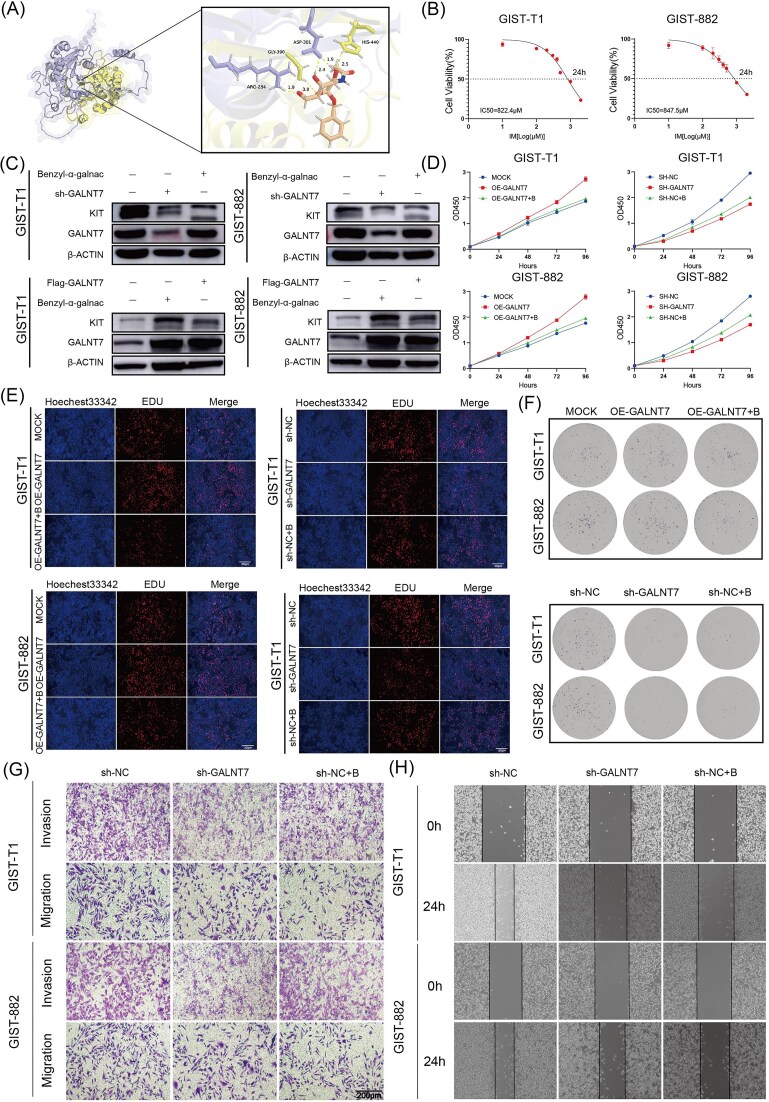
Benzyl-α-GalNAc suppresses GALNT7-driven malignant phenotypes of GIST cells *in vitro*. (**A**) Three-dimensional molecular docking model showing the predicted binding of benzyl-α-GalNAc to GALNT7, suggesting a potential direct interaction between the inhibitor and the GALNT7 protein. (**B**) Dose-response curves showing the cytotoxic effects of benzyl-α-GalNAc on GIST-T1 and GIST-882 cells after 24 hours of treatment. IC50 values were calculated based on cell viability assays. (**C**) Benzyl-α-GalNAc sustains KIT protein suppression regardless of GALNT7 expression levels by Western blot. (**D–F)** Proliferation of GALNT7-modulated GIST cells upon benzyl-α-GalNAc treatment assessed by CCK-8, colony formation, and EDU assay. (**G, H**) Transwell and wound healing assays in *GALNT7*-overexpressing or knockdown GIST-T1 and 882 cells under benzyl-α-GalNAc treatment. OE-GALNT7, GALNT7 overexpression lentivirus; Mock, normal control lentivirus; shGALNT7, GALNT7 shRNA lentivirus, shNC, negative control shRNA lentivirus. Data are presented as mean ± SEM. *P < 0.05, **P < 0.01, ***P < 0.001, ****P < 0.0001.

Building upon reported evidence regarding the therapeutic potential of the O-glycosylation inhibitor benzyl-α-GalNAc in liver lipid deposition and colorectal cancer [46, 47], we employed molecular docking to demonstrate for the first time that this small molecule specifically binds to the subdomain of GALNT7 (Fig. 7A and supplementary Fig. 7A). To investigate its functional impact in GIST, we first determined the half-maximal inhibitory concentration (IC50) of benzyl-α-GalNAc in GIST cell lines using dose-gradient assays (Fig. 7B). Subsequent mechanistic studies revealed that benzyl-α-GalNAc effectively reversed GALNT7-mediated regulation of KIT protein stability, as consistently validated in both *GALNT7*-overexpressing and knockdown cell models (Fig. 7C). Functional experiments, including cell viability assays, colony formation assays, and EDU assay, confirmed that benzyl-α-GalNAc treatment significantly suppressed the enhanced proliferative capacity induced by GALNT7 overexpression (Fig. 7D–F and supplementary Fig. 7B and C). Moreover, wound healing (Fig. 7G and supplementary Fig. 7D) and Transwell migration and invasion assays (Fig. 7H and supplementary Fig. 7E) demonstrated that benzyl-α-GalNAc effectively inhibited the migratory and invasive capacities of GIST cells driven by GALNT7. Our study systematically elucidates the molecular mechanism by which benzyl-α-GalNAc targets the GALNT7–KIT glycosylation axis to suppress malignant progression in GIST. These findings not only confirm the specific inhibitory effect of benzyl-α-GalNAc on GALNT7 function but also highlight its therapeutic potential in reversing proliferative, migratory, and invasive phenotypes of GIST, thereby providing novel experimental evidence and promising strategies for targeted therapy in GIST.

### Targeting GALNT7 with benzyl-α-GalNAc inhibits GIST tumor growth and metastasis *in vivo*

**Figure 8:**
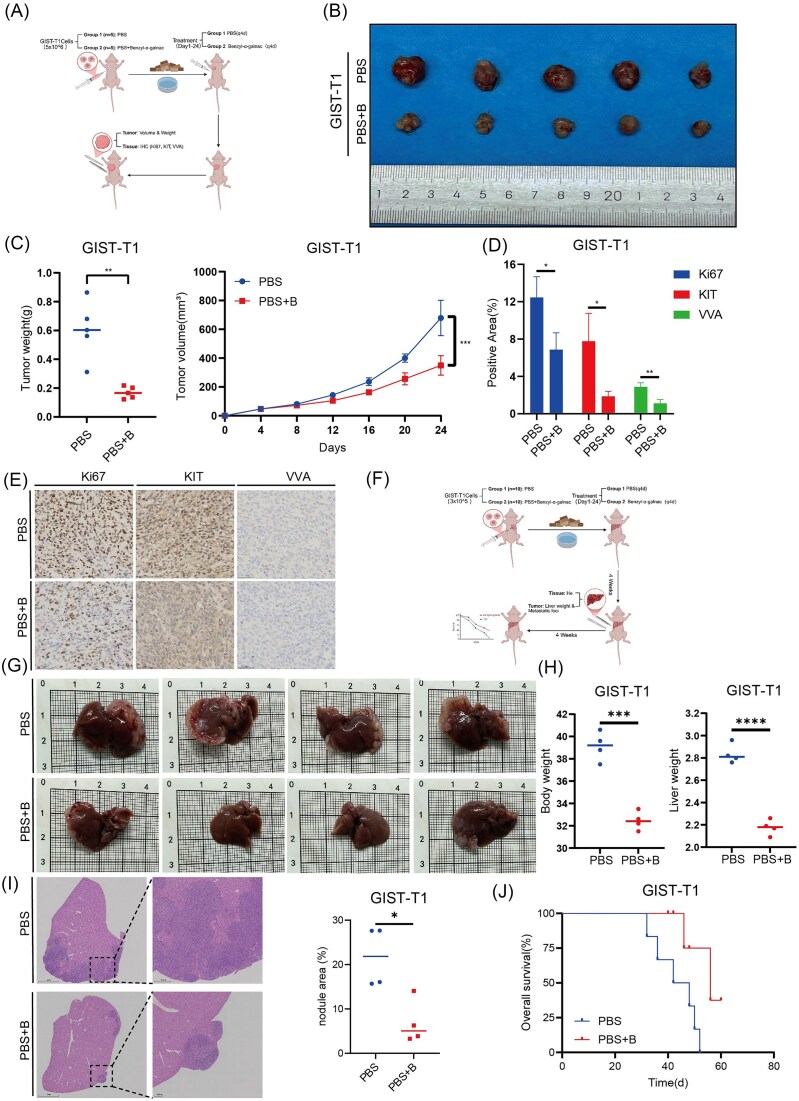
Benzyl-α-GalNAc inhibits GIST tumor growth and liver metastasis *in vivo*. (**A**) Schematic illustration of the subcutaneous xenograft experiment used to evaluate the anti-tumor effect of benzyl-α-GalNAc *in vivo*. (**B**) Representative images of subcutaneous xenograft tumors from PBS-treated and benzyl-α-GalNAc-treated mice. (**C**) Tumor growth curves showing that benzyl-α-GalNAc treatment significantly reduced xenograft tumor volume over time. (**D**) Quantification of tumor weights at the endpoint, demonstrating reduced tumor burden in the benzyl-α-GalNAc-treated group. (**E**) Representative immunohistochemical staining of xenograft tumor tissues for Ki67, KIT, and VVA (bar = 50 µm). (**F**) Schematic illustration of the liver metastasis model used to assess the effect of benzyl-α-GalNAc on metastatic progression *in vivo*. (**G**) Representative images of hepatic xenograft tumors. (**H**) Quantification of mouse body weight and liver weight in the liver metastasis model. (**I**) H&E-stained liver metastases and lesional regions derived from GIST-T1 cell. Scale bar, 250 μm. (**J**) Kaplan–Meier survival curve for tumor-bearing mice (*n* = 6). PBS + B, PBS + benzyl-α-GalNAc. Data are presented as mean ± SEM. *P < 0.05, **P < 0.01, ***P < 0.001, ****P < 0.0001.

To evaluate the inhibitory effect of benzyl-α-GalNAc on GIST tumorigenesis *in vivo*, GIST-T1 cells were subcutaneously inoculated into two groups of nude mice. One group was administered benzyl-α-GalNAc (1 mg/mouse; subcutaneously; once daily) every four days, while the control group was administered an equal volume of PBS. Tumor volume was measured every four days. We found that mice treated with benzyl-α-GalNAc exhibited significantly smaller tumor sizes and lower tumor weights compared to the control group, indicating that benzyl-α-GalNAc suppresses GIST tumor growth *in vivo* (Fig. 8A–C). Consistent with these findings, immunohistochemical analysis of dissected tumor tissues revealed markedly reduced expression levels of the proliferation marker Ki67, as well as KIT and VVA, in the benzyl-α-GalNAc-treated group (Fig. 8D and E). Similarly, in the liver metastasis mouse model, identical grouping and treatment protocols were applied. Compared to the control group, the benzyl-α-GalNAc-treated group exhibited significantly lower body weight in nude mice and reduced mass of the lesioned livers (Fig. 8G–I), along with a marked decrease in both the number and size of hepatic metastatic lesions, ultimately leading to a significant extension of overall survival in the animals. In addition, in tumor-bearing mice not treated with benzyl-α-GalNAc, tumor burden was negatively correlated with survival time (Fig. 8J). These results demonstrate that benzyl-α-GalNAc attenuates the malignant phenotype of GIST cells, likely through partial inhibition of GALNT7-mediated O-glycosylation of KIT.

## Discussion

As a key member of the GALNTs family, GALNT7 is not only regulated by multiple transcription factors but also modulates O-glycosylation levels to promote tumor growth in prostate cancer, indicating its close association with tumor progression [48–50]. The specific role of GALNT7 in GIST, a tumor with distinct driver mutations, remains unexplored, in contrast to its relatively well-defined mechanisms in cancers such as prostate cancer. Significant advances have been made in recent years in understanding the pathogenesis and treatment of GIST, particularly through the application of TKIs targeting the KIT/PDGFRA signaling pathway [51]. However, primary and secondary drug resistance remain major clinical challenges [52], highlighting the urgent need to explore novel molecular drivers and alternative therapeutic strategies for GIST.

Through multiomics analysis, this study reveals for the first time that O-GalNAc glycosylation is widely activated in GIST and systematically demonstrates the critical oncogenic role of the glycosyltransferase GALNT7 in GIST malignant progression via its modification of the KIT protein. Additionally, we validate the therapeutic potential of a novel inhibitor, benzyl-α-GalNAc, targeting this axis. By investigating the correlation between GIST and O-glycosylation and integrating transcriptomic, proteomic, and single-cell RNA sequencing data, this study provides the first multidimensional evidence of a positive correlation between O-glycosylation and GIST pathological risk stratification. Furthermore, trajectory analysis at the single-cell level revealed that O-glycosylation scores synchronously increase with CNV levels in a fibroblast subpopulation of higher malignancy, suggesting that aberrant O-glycosylation may be a key molecular event in the early pathogenesis and progression of GIST. Among numerous O-glycosylation-related genes, GALNT7 was identified as a core driver through rigorous bioinformatic screening and clinical data validation.

Our *in vitro* and *in vivo* experiments demonstrate that GALNT7 promotes malignant phenotypes in GIST, and given the central role of KIT in GIST pathogenesis [6], we further discovered and confirmed for the first time that GALNT7 directly interacts with the KIT protein and catalyzes its O-GalNAc glycosylation. This modification directly enhances KIT protein stability, delays its degradation, and consequently leads to sustained activation of the downstream PI3K/AKT and MAPK/ERK signaling pathways. This finding not only reveals a novel post-transcriptional regulatory mechanism that “reinforces” KIT oncogenic activity beyond gene mutations but also, to some extent, explains the aberrantly active KIT signaling pathway in high-risk GIST, providing a new biomarker for molecular subtyping and prognosis diagnosis of GIST. However, the specific short O-glycan structures catalyzed by GALNT7 and their precise biological functions in the malignant progression of GIST remain elusive. Moreover, the exact amino acid residue on the KIT protein modified by O-GalNAc glycosylation has not been mapped. Identifying this specific glycosylation site is essential for elucidating the regulatory mechanism at an atomic resolution.

In summary, this study not only establishes O-GalNAc glycosylation as a novel molecular signature of GIST but also elucidates the detailed mechanism by which GALNT7 stabilizes the KIT oncoprotein via O-GalNAc glycosylation, thereby driving the malignant progression of GIST. It positions GALNT7 as a novel prognostic marker and a potential therapeutic target. More importantly, the results indicate that targeting the GALNT7–KIT glycosylation axis using benzyl-α-GalNAc represents a highly promising new strategy for overcoming GIST resistance to existing targeted therapies.

## Ethics statement

This study received approval from the Ethics Committee of the First Affiliated Hospital of Zhengzhou University (Approval No. 2024-KY-1592–001) and the Institutional Animal Welfare and Use Committee of the Henan Institute of Medical and Pharmaceutical Sciences (Approval No. 2025-YYY-052), and written informed consent was obtained from each participant.

## Supplementary Material

pbag016_Supplemental_File
