# Supplementary material for: GALNT7 promotes the malignant progression of gastrointestinal stromal tumors by regulating KIT O-GalNAc glycosylation
Source: Precis Clin Med. 2026 May 27;9(2):pbag016. doi: 10.1093/pcmedi/pbag016 (PMC13256011; doi:10.1093/pcmedi/pbag016)
Supplement: pbag016_Supplemental_File [file pbag016_supplemental_file.docx]

**Supplementary Data**

**Supplementary Table 1** The primer sequences.

| Genes | Forward Primer | | Reverse Primer |
| --- | --- | --- | --- |
| GALNT7 | TTACGCAGTTTGCTGGTGGT | | CTGTCTTCCCTCATCCTGCTC |
| KIT | GCACAATGGCACGGTTGAAT | | GGTGTGGGGATGGATTTGCT |
| β-actin | TGGCACCCAGCACAATGAA | CTAAGTCATAGTCCGCCTAGAAGCA | |

**Supplementary Table 2** Correlation between GALNT7 score in GIST and clinicopathological factors.

| Clinical factors |  | GALNT7 | | *P* Value |
| --- | --- | --- | --- | --- |
|  |  | low | high |  |
| **sex** | male | 28 | 24 | 0.461 |
|  | female | 32 | 36 |  |
| **age** | <60 | 37 | 30 | 0.198 |
|  | >=60 | 23 | 30 |  |
| **location** | stomach | 34 | 27 | 0.064 |
|  | duodenum | 6 | 4 |  |
|  | small intestine | 13 | 20 |  |
|  | colorectum | 5 | 1 |  |
|  | other | 2 | 8 |  |
| **medication** | yes | 26 | 38 | 0.028 |
|  | no | 34 | 22 |  |
| **risk** | high | 20 | 40 | 0.002 |
|  | middle | 23 | 11 |  |
|  | low | 15 | 8 |  |
|  | very low | 2 | 1 |  |
| **recurrence** | yes | 4 | 16 | 0.003 |
|  | no | 56 | 44 |  |


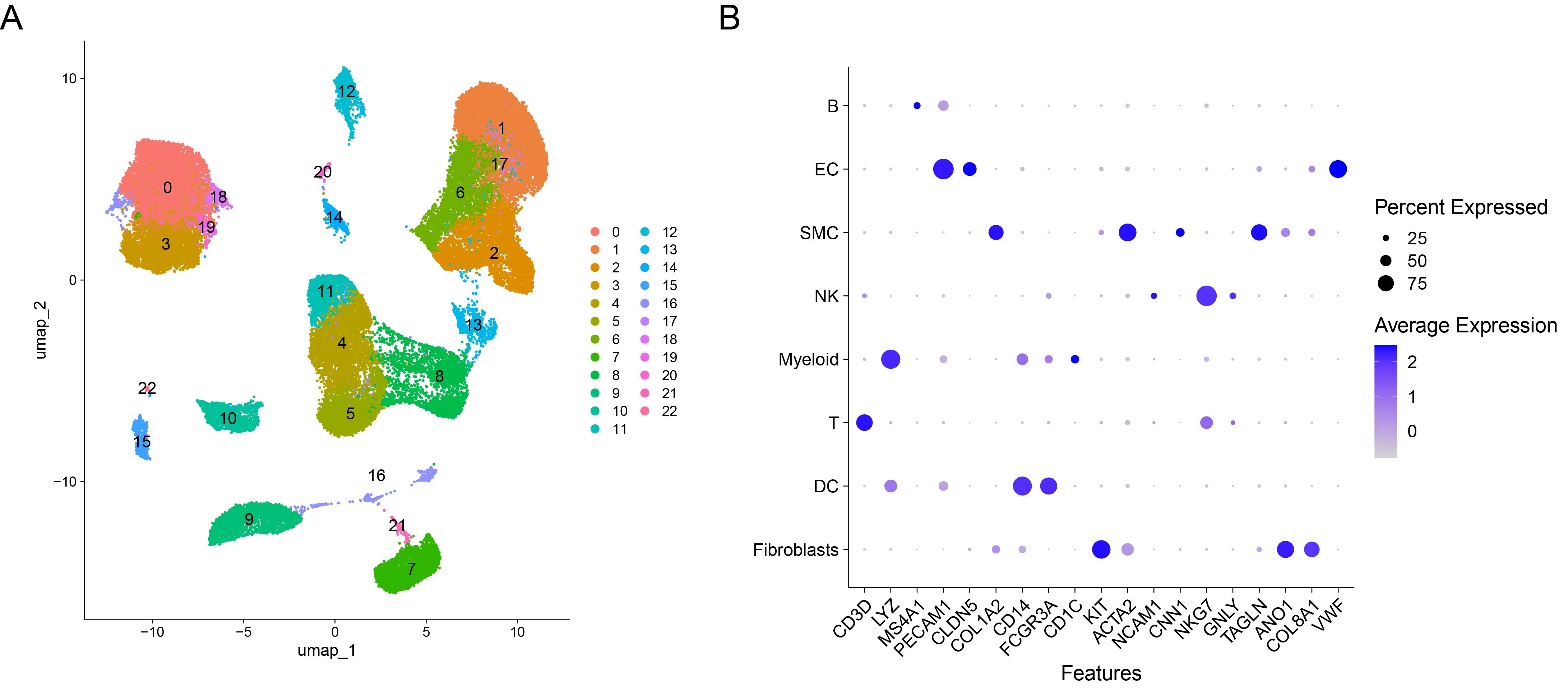


**Supplementary Figure** **1** Clustering and marker expression for scRNA-seq data from GSE254762. (**A**) UMAP visualization of all cell population, colored by cluster identity. (**B**) Dot plot visualizing the expression of marker genes across distinct cell types.


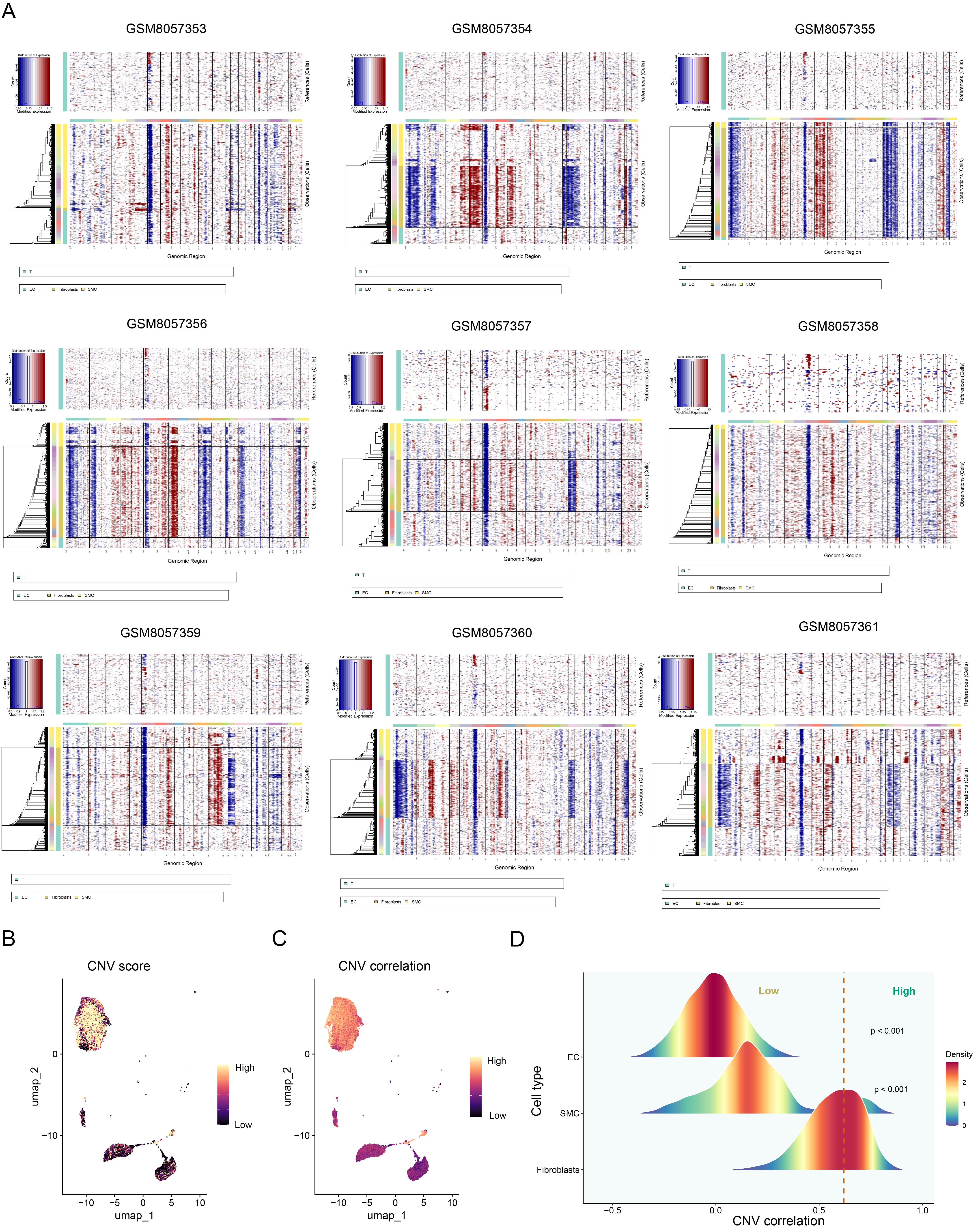


**Supplementary Figure** **2** InferCNV analysis of stromal cells. (**A**) Hierarchical heatmap depicting large-scale CNVs in SMC, EC and fibroblasts from each sample. (**B**) UMAP plot showing the distribution of CNV score in stromal cells. (**C**) UMAP plot showing the distribution of CNV correlation in stromal cells. (**D**) Ridgeline plots illustrating the distribution of CNV correlation across stromal cells. Cells were grouped based on CNV correlation, with the red dashed line demarcating the specific threshold value.


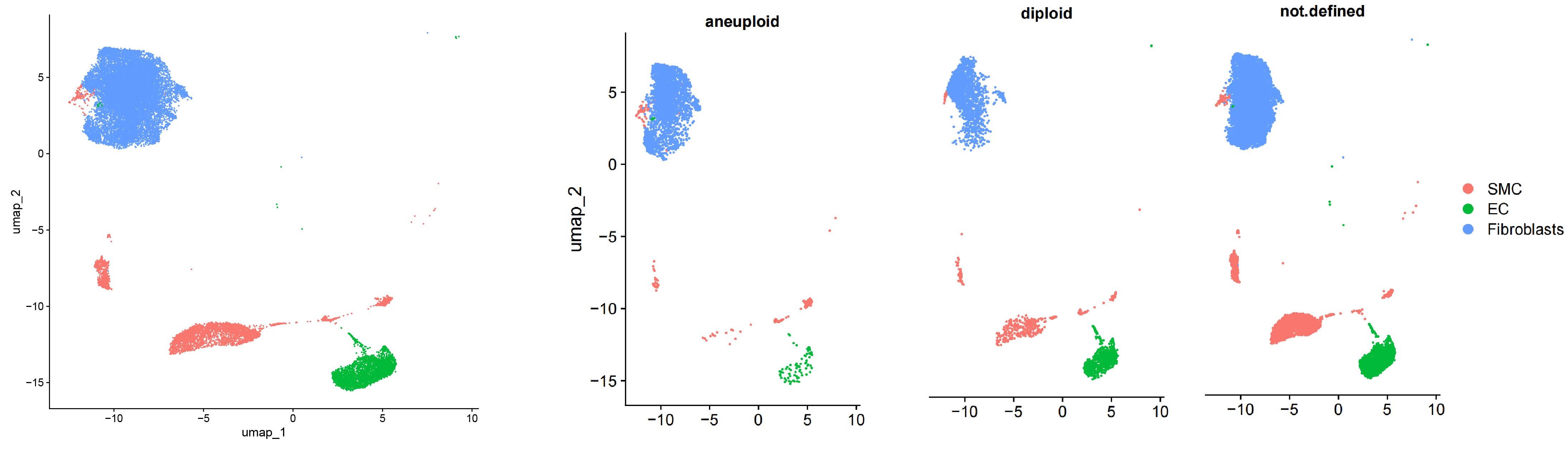


**Supplementary Figure 3** CopyKAT analysis of stromal cells.


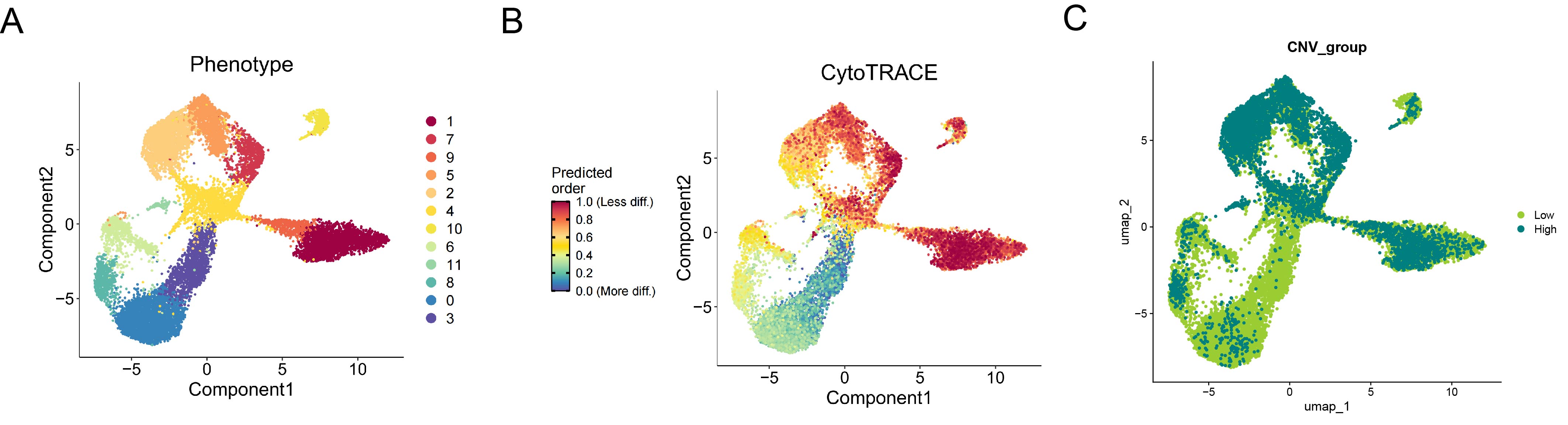


**Supplementary Figure** **4** Trajectory analysis of fibroblasts. (**A**) UMAP visualization of fibroblasts, colored by cluster identity. (**B**) UMAP plot illustrating the distribution of CytoTRACE scores within fibroblasts, where dark-blue represents low scores (more differentiation) and dark-red represents high scores (less differentiation). (**C**) UMAP plot showing the distribution of high and low CNV groups in fibroblasts.


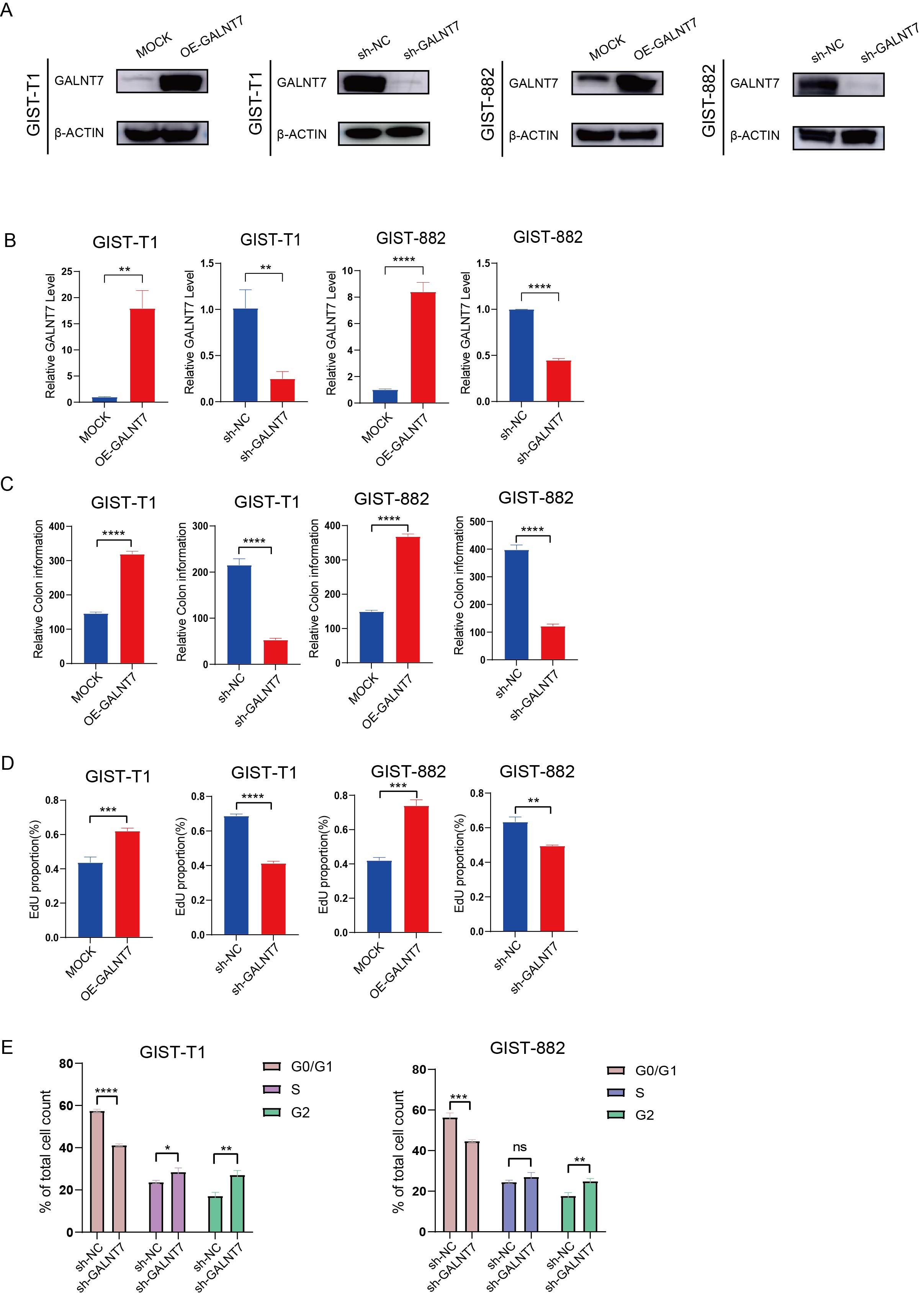


**Supplementary Figure 5** GALNT7 regulates the Proliferation of GIST Cells both *in vitro* and i*n vivo*. (**A**) Overexpression and knockdown of GALNT7 in GIST-T1 and GIST-882 were analyzed by Western blot. (**B**) Overexpression and knockdown of GALNT7 in GIST-T1 and GIST-882 were analyzed by RT-qPCR. (**C**) Statistical results of the colony formation assay. (**D**) Statistical results of the EDU assay. (**E**) Statistical results of the flow cytometric analysis of cell cycle. **P* < 0.05, ***P* < 0.01, ****P* < 0.001, *****P* < 0.0001.


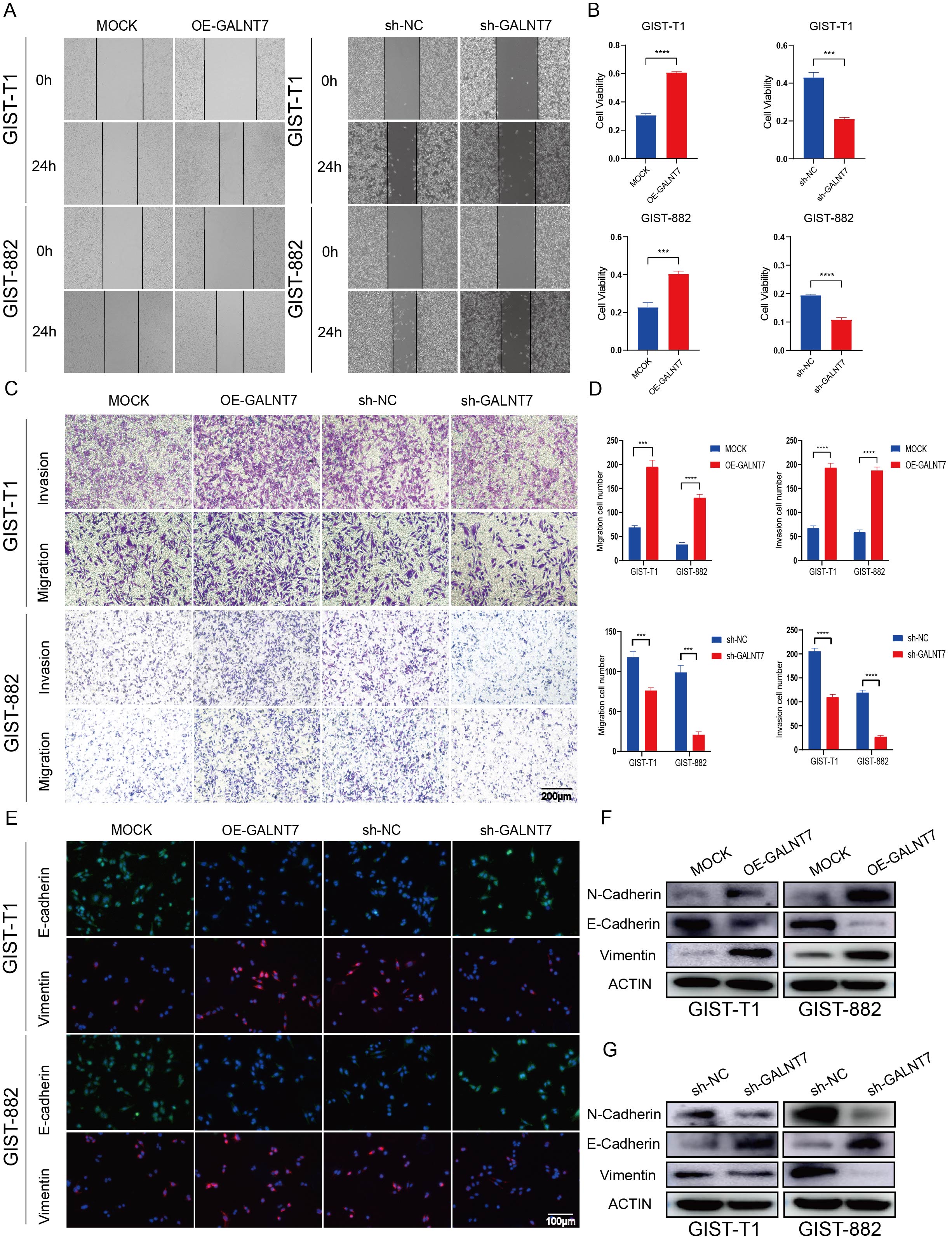


**Supplementary Figure** **6** GALNT7 regulates the migration and invasion of GIST cells. (**A-B**) Wound Healing Assay. The wounded areas were photoimaged immediately after wounding and 24 h post-wounding. Wound healing area (%) = (0 h Wound area -24h Wound area)/0 h Wound area×100%. (**C-D**) Transwell migration and invasion assay (bar = 200µm). (**E**) Immunofluorescence analysis (bar = 100µm). (**F-G**) Expression of metastasis-related markers after GALNT7 overexpression or knockdown by Western blot. OV GALNT7 overexpression lentivirus, Mock normal control lentivirus, shRNA GALNT7 shRNA lentivirus, shNC negative control shRNA lentivirus. **P* < 0.05, ***P* < 0.01, ****P* < 0.001, *****P* < 0.0001.


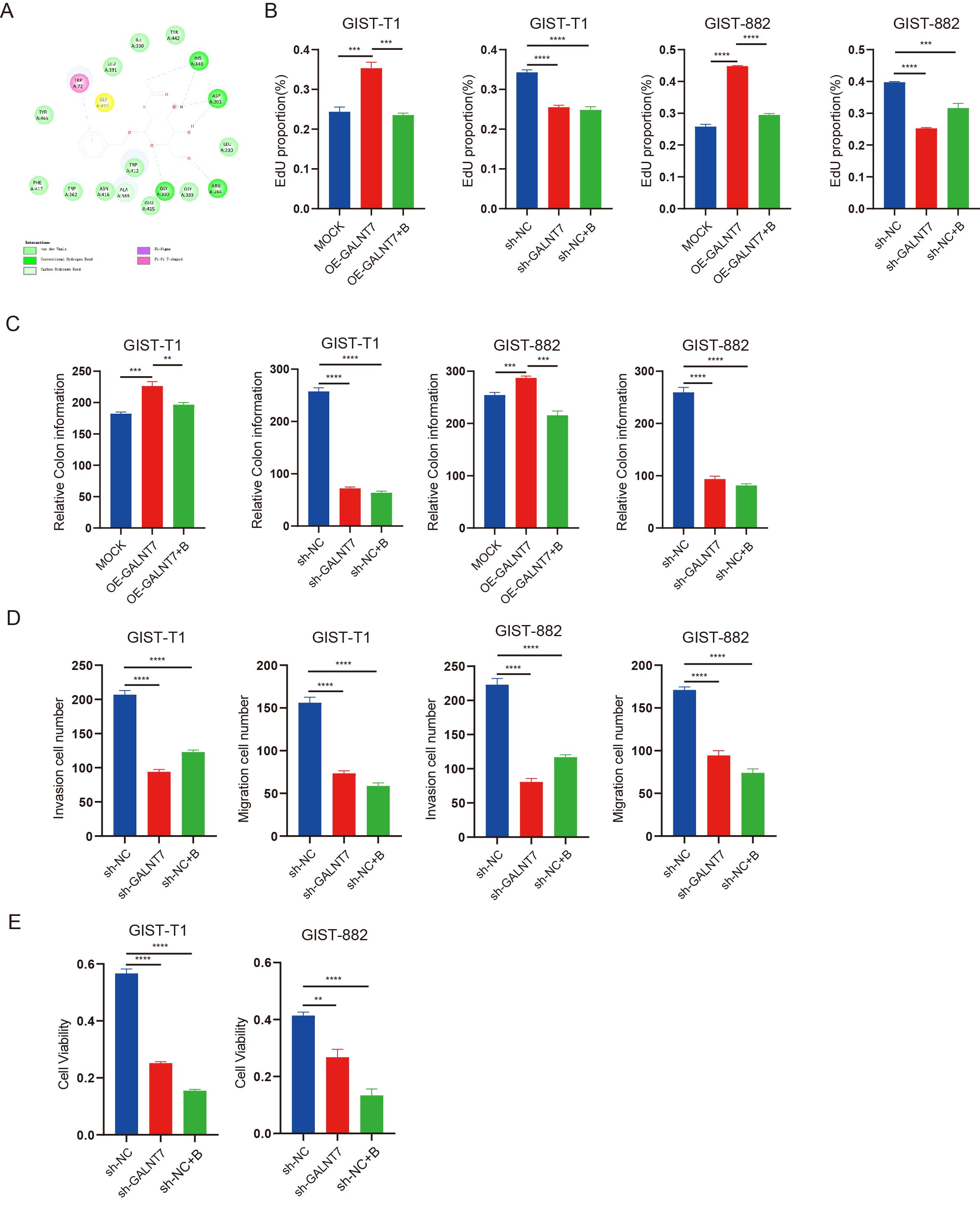


**Supplementary Figure 7** Targeting the GALNT7-KIT Glycosylation Axis with Benzyl-α-GalNAc Attenuates Malignant Progression in GIST. (**A**) 2D docking model of Benzyl-α-GalNAc with the GALNT7 protein. (**B**) Statistical results of the EDU assay. (**C**) Statistical results of the colony formation assay. (**D**) Statistical results of the transwell migration and invasion assay. (E) Statistical results of the wound Healing Assay. **P* < 0.05, ***P* < 0.01, ****P* < 0.001, *****P* < 0.0001.
